# Advancing Precision Medicine in PDAC: An Ethical Scoping Review and Call to Action for IHC Implementation

**DOI:** 10.3390/cancers17121899

**Published:** 2025-06-06

**Authors:** Lyanne A. Delgado-Coka, Lucia Roa-Peña, Andrew Flescher, Luisa F. Escobar-Hoyos, Kenneth R. Shroyer

**Affiliations:** 1 Department of Pathology, Renaissance School of Medicine, Stony Brook University, Stony Brook, NY 11794, USA; luisa.escobar-hoyos@yale.edu (L.F.E.-H.);; 2 Program of Public Health and Department of Preventative Medicine, Renaissance School of Medicine, Stony Brook University, Stony Brook, NY 11794, USA; andrew.flescher@stonybrook.edu; 3 Department of Pathology, School of Medicine, Universidad Nacional de Colombia, Bogotá 110110, Colombia; 4 Department of Therapeutic Radiology, Yale University, New Haven, CT 06510, USA; 5 Department of Molecular Biophysics and Biochemistry, Yale University, New Haven, CT 06520, USA; 6 Division of Oncology-Medicine-Oncology, Yale University, New Haven, CT 06510, USA

**Keywords:** pancreatic ductal adenocarcinoma, PDAC, immunohistochemistry, IHC, molecular subtyping, ethical considerations, scoping review, autonomy, beneficence, non-maleficence, justice

## Abstract

Pancreatic ductal adenocarcinoma (PDAC) presents significant challenges in diagnosis, prevention, and treatment. Predictive biomarkers offer the potential to revolutionize clinical management, particularly in the preoperative setting, but their implementation requires careful consideration of ethical implications. This scoping review analyzes the ethical landscape of using immunohistochemistry (IHC) for molecular subtyping in PDAC, focusing on its utility, accessibility, and potential impact on patient care. We conducted a systematic literature search in the PubMed, Scopus and Google Scholar databases (2015–2025) using COVIDENCE, which identified 130 references. Of these, 79 were reviewed in a full-text format, and 9 ultimately met the inclusion criteria for our analysis. IHC offers several advantages as a companion diagnostic tool. It is relatively inexpensive, widely available in most pathology laboratories, and can be readily integrated into existing clinical workflows. This contrasts with more complex molecular subtyping methods, such as gene expression profiling, which can be costly, require specialized equipment and expertise, and may not be readily accessible in all clinical settings. Furthermore, accurate analysis of gene expression requires the localized targeting of individual cells; therefore, digesting the sample for bulk analysis would be less informative than using spatial localization techniques such as IHC. Because biomarker regulation can occur at the level of transcription or translation, protein-level assessment via IHC is often more accurate than mRNA analysis. Standardized IHC protocols for biomarker assessment are therefore essential for translating the molecular subtyping of PDAC into clinically actionable treatment strategies, especially for aggressive subtypes like basal-like tumors. This readily deployable IHC-based approach can optimize therapy selection, maximizing patient benefits and minimizing exposure to ineffective and potentially toxic treatments. This review critically analyzes the ethical dimensions of this method, grounded in the principles of autonomy, beneficence, non-maleficence, and justice. The review urges the medical community to fully utilize the potential of IHC-driven molecular subtyping to improve outcomes in PDAC, while ensuring equitable and responsible access to the benefits of precision oncology for all patients.

## Simple Summary

Pancreatic cancer is among the deadliest cancers, with few patients qualifying for surgery, the only potential cure. For everyone else, treatments remain limited, and survival rates are disturbingly low. While expensive genetic tests only help a small fraction of patients, this review examines how a widely available and affordable laboratory test—immunohistochemistry (IHC)—could transform cancer care by matching patients with the most effective treatments. Instead of relying on a one-size-fits-all treatment approach for pancreatic cancer, using IHC to detect specific biomarkers can help guide clinicians in tailoring therapies to each patient’s unique tumor, potentially improving outcomes while minimizing side effects. We analyze the ethical imperative to expand clinical trials of IHC-based testing, ensuring this life-saving technology reaches all communities, regardless of their resources or location. By implementing this cost-effective approach in hospitals worldwide, we could dramatically improve how we treat pancreatic cancer, saving lives and reducing healthcare expenditure while offering hope to patients and families facing this aggressive disease. This practical solution could become a model for delivering more equitable and effective cancer care across the healthcare system.

## 1. Introduction

### 1.1. Highlighting the Persistent Challenge of Pancreatic Cancer

Pancreatic ductal adenocarcinoma (PDAC) remains a formidable challenge in oncology, characterized by a dismal prognosis and limited treatment options. Notably, despite advances in cancer care, only a minority (approximately 20%) of PDAC patients present with resectable disease, the only potentially curative intervention [1]. In contrast, for the remaining majority, systemic therapies offer limited long-term survival benefit [2]. This stark reality underscores the urgent need for innovative diagnostic and therapeutic strategies to improve outcomes for all PDAC patients, regardless of resectability. The aggressive nature of PDAC [3,4] and its projected rise as a leading cause of cancer-related death worldwide further underscore the pressing need for innovative approaches to treatment and patient management.

**Figure 1:**
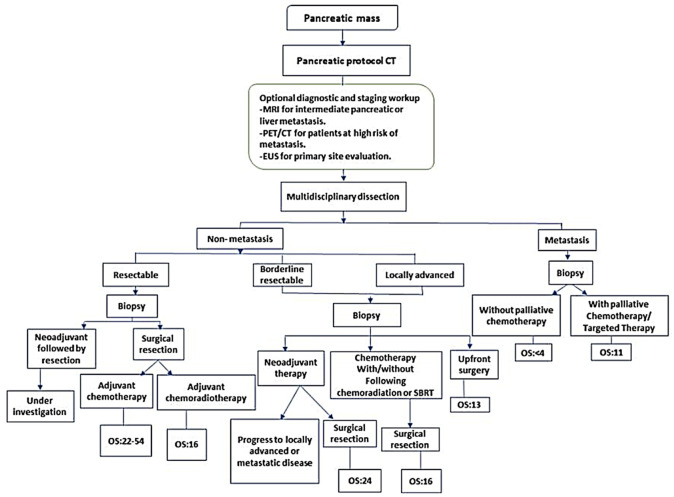
Current diagnostic and therapeutic pathways for staging, tumor response evaluation, and management of borderline or resectable PDAC. CT, computed tomography; EUS, endoscopic ultrasound; MRI, magnetic resonance imaging; PET, positron emission tomography; SBRT, stereotactic body radiation therapy. NCCN guidelines recommend that biopsy is not required for proof of malignancy prior to surgical resection and should not delay surgical resection in patients with high clinical suspicion of pancreatic cancer. Reprinted from Bugazia, Doaa et al. [5].

The current clinical management of PDAC heavily relies on the detection of incidental lesions via imaging studies, typically performed using computed tomography (CT) or magnetic resonance imaging (MRI conducted for unrelated medical issues). This is invariably followed by tissue biopsy [5] for histopathological confirmation and the assessment of tumor resectability (Figure 1) [6].

However, while often combined with neoadjuvant chemotherapy, this standard approach yields disappointingly low long-term survival [7]. This limitation stems from the fact that routine histopathology does not fully capture the complex biological behavior of PDAC, including its aggressiveness, chemoresistance, and metastatic potential. Additionally, the inherent chemoresistance of PDAC, coupled with significant genetic heterogeneity and a dense stromal microenvironment [8], further complicates effective treatment. As highlighted by Hilmi et al., PDAC exhibits both inter- and intratumoral heterogeneity, encompassing variations between patients and within individual tumors [9]. This heterogeneity manifests histologically, with diverse architectures like squamous/non-gland-forming (associated with the basal-like subtype) and glandular/gland-forming (associated with the classical subtype) varieties coexisting within the same tumor. Existing interventions, including surgery, radiation, chemotherapy, immunotherapy, and targeted therapies, often only provide marginal improvements in overall survival. This underscores the critical need to identify and validate novel biomarkers to enhance personalized treatment strategies.

Transcriptomic subtyping has emerged as a valuable tool for predicting treatment response and tailoring therapy based on individual tumor biology [10,11]. Specifically, some studies demonstrate that basal-like PDAC carries a poorer prognosis and greater chemoresistance compared to the classical subtype, highlighting the importance of subtype-specific treatment strategies [12,13,14,15]. However, bulk transcriptomic analyses, while informative, are costly, difficult to implement clinically, and struggle to fully capture the complexities of intratumoral heterogeneity due to their inherent averaging effect across diverse cell populations. This averaging effect obscures critical information about the spatial distribution and interactions of different cell types within the tumor. As a result, the limitations of bulk transcriptomics have led to the proposal of continuous molecular gradients to better represent tumor characteristics, but these still lack information on intratumoral heterogeneity [9]. While single-cell studies offer higher resolution and have revealed the co-occurrence of classical and basal-like subtypes within individual tumors (hybrid/mixed tumors), including an intermediate phenotype with the co-expression of both classical and basal markers [16], their widespread clinical application remains limited by cost, complexity, and the need for specialized expertise. The application of deep learning models to H&E staining to identify intermediate tumors—where cells cannot be clearly assigned to either subtype—further emphasizes the complexity of PDAC heterogeneity and the need for more robust and accessible methods to characterize it [17,18]. Biomarker-driven testing is thus becoming a cornerstone of personalized medicine in PDAC [19]. By identifying specific tumor characteristics, such as basal subtype, clinicians can move away from a one-size-fits-all approach towards tailored therapies, maximizing efficacy and minimizing treatment-related toxicity [20]. Additionally, trials like SWOG S1505 reveal the difficulty of assessing chemotherapy efficacy in unselected populations, thus reinforcing the need for genomically driven precision and patient stratification. Consequently, integrating biomarker-enriched designs into neoadjuvant settings is therefore crucial for advancing precision medicine in PDAC [21].

### 1.2. The Promise of IHC in the Era of Advanced Molecular Technologies

While next-generation sequencing (NGS), artificial intelligence, and multi-omics have revolutionized our understanding of tumor biology, their complexity and cost limit widespread clinical application [22]. Although NGS accessibility and affordability are increasing, barriers to broader adoption persist, including investment costs, limited reimbursement, infrastructure limitations, centralization in specialized centers, and the lack of standardized guidelines. While the further research and development of targeted therapies, based on actionable mutations identified by NGS, remain crucial, immunohistochemistry (IHC) offers a pragmatic solution. As emphasized by Hilmi et al. (2025), IHC provides a cost-effective and readily accessible method for analyzing individual tumor cells while preserving their spatial context, enabling the more precise assessment of intratumoral heterogeneity compared to bulk transcriptomics or H&E staining alone [9]. This spatial resolution, which allows for the analysis of individual cells and their localization, is critical for understanding the complex interplay between different cell populations within the tumor microenvironment and for developing more accurate diagnostic and prognostic tools [23,24]. Furthermore, IHC avoids the averaging effect of bulk transcriptomics and the complexities of single-cell analysis, providing a practical and efficient approach to characterizing tumor heterogeneity, particularly when applied on a larger scale, where current methods are lacking [9]. To translate these theoretical advantages into clinical practice, IHC provides a readily available, cost-effective, and easily integrated method for biomarker detection in routine clinical practice, particularly benefiting those patients deemed unresectable, whose treatment goal is palliative care. Developing an IHC-based biomarker assay to guide neoadjuvant and adjuvant chemotherapy selection is critical. However, despite IHC’s practical advantages, one key challenge remains, namely, identifying robust panels of IHC biomarkers that accurately predict treatment benefits and comprehensively capture the complexities of intratumoral heterogeneity.

To address these challenges and develop a robust IHC-based tool for predicting treatment response in PDAC, careful biomarker selection is essential, particularly given the limitations of traditional RNA-based approaches in distinguishing tumor-specific markers from stromal signatures. Building on established molecular knowledge, when selecting appropriate biomarkers for IHC analysis, ideal biomarkers should (i) be expressed by tumor cells only and not by stromal cells, (ii) have available antibodies that produce staining compatible with routine clinical use (considering intensity, ease of interpretation, and robustness), and (iii) be informative of treatment response independently of other tumor characteristics. Bulk transcriptomic analysis can misrepresent gene expression differences in PDAC subtypes. For instance, while *KRT6A* is associated with the epithelial component in both basal-like and classical tumors, its expression appears higher in basal-like tumors, simply due to their increased cellularity. This difficulty in interpreting true subtype-specific expression limits the clinical utility of bulk transcriptomics in PDAC [9]. Previous studies, along with our own findings, support the need for a panel of markers rather than single markers to address PDAC heterogeneity [12]. For example, Ragavhan et al. (2021) recently proposed a multiplex immunofluorescence panel including six markers (CLDN18.2, TFF1, GATA6, KRT17, KRT5, and S100A2) [16]. Several clinical trials like the PRODIGE-104 (NeoPREDICT) phase II trial aim to personalize neoadjuvant chemotherapy according to transcriptomic signatures in borderline resectable PDAC. To enhance the clinical utility of this trial, the IHC staining of a classical marker (e.g., CLDN18) and two specific basal markers (S100A2 and MUC16) were incorporated into the ancillary studies. This combined approach promises to refine patient prognosis and facilitate the implementation of these markers in routine clinical practice. Similarly, the ongoing NeoPANCOne phase II trial (NCT04472910) is evaluating the value of GATA6 (assessed by FISH and IHC) in predicting chemotherapy resistance in resectable PDAC [25]. Facilitating the assessment of tumor heterogeneity through IHC could also prove valuable in evaluating treatment response in trials of novel therapies that modulate PDAC phenotypes, such as epigenetic therapies [26,27].

In resectable PDAC, the indiscriminate administration of neoadjuvant chemotherapy can be detrimental, potentially leading to disease progression or toxicity that precludes curative surgery. Given the limited progress in developing robust predictive biomarkers, current patient selection for neoadjuvant chemotherapy relies on high-risk clinical and radiographic features, such as the detection of suspicious lymphadenopathy/metastases via imaging and elevated CA19-9 levels. IHC offers a practical approach to detecting prognostic and predictive biomarkers, holding immense potential for advancing personalized medicine in diverse healthcare settings. Leveraging IHC-based biomarkers in preoperative clinical trials allows for refined risk stratification and informs decisions regarding primary systemic therapy by identifying markers of therapy resistance or sensitivity. This personalized approach not only improves patient outcomes but also minimizes treatment-related toxicity and improves quality of life for patients and their families. Given the limitations of current PDAC management and the potential of IHC, this scoping review systematically evaluates the existing literature on IHC-based biomarkers in PDAC. This review will perform the following tasks:

• Delineate the current landscape of predictive IHC biomarkers in PDAC, highlighting successes and challenges.
• Analyze the ethical implications of using IHC to guide treatment decisions, focusing on the principles of autonomy, beneficence, non-maleficence, and justice.
• Assess how IHC-based approaches align with clinical needs and patient welfare.

By providing a comprehensive overview of the evidence, this review aims to inform future public health policies and clinical practices, ultimately promoting the effective integration of IHC into personalized medicine strategies for combating pancreatic cancer.

## 2. Methods

A scoping review was conducted between November 2024 and February 2025 using the PRISMA-ScR (Preferred Reporting Items for Systematic reviews and Meta-Analyses extension for Scoping Reviews) guidelines. A protocol was developed for the scoping review. This consisted of the following steps: exploratory investigation of the literature; the identification of research aims; the formulation of eligibility criteria; database searches; the screening and identification of studies; and extraction and charting of data.

**Initial Search:** To gain insight into the topic area, an exploratory investigation of the literature was undertaken, employing broad criteria across multiple databases. This investigation informed the selection of keywords for the searches and formulation of the eligibility criteria.

**Search Strategy and Screening:** The following electronic databases were searched: PubMed, Google Scholar, and Scopus. The most recent search was undertaken on 26 March 2025. Searches were conducted for each database from 2015 to the present. Keywords and Medical Subject Heading (MeSH) terms encompassed “Pancreatic Ductal Adenocarcinoma”, “PDAC”, “Transcriptomics”, “Gene Expression Profiling”, “Immunohistochemistry”, “Molecular Subtyping”, “Precision Medicine”, “Biomarkers”, “Predictive”, “Treatment Outcome”, and “Clinical Utility”. A total of 79 articles were identified from the search. The references of the articles were scanned to identify any additional relevant articles; no additional studies were included. Duplicates were removed.

**Eligibility Criteria:** Studies meeting the following criteria were included: the population criterion referred to patients diagnosed with PDAC; the concept criterion referred to studies investigating the ethical implications of IHC-based molecular subtyping in PDAC. This includes evaluations of IHC utility, accessibility, cost-effectiveness, and the impact on treatment decisions and patient outcomes. Studies were excluded if they were not related to PDAC; did not employ IHC-based molecular subtyping in PDAC; lacked sufficient clinical outcome data related to identified subtypes; or were reviews offering no novel insights (with some flexibility for context-providing narrative reviews). Studies on adults with all stages of PDAC were included. Studies in which biomarkers were not assessed as an outcome were excluded. All types of clinical trials were included to maximize the comprehensive nature of the search.

**Study Selection:** Two reviewers (L.A.D.-C, L.R.-P) conducted an initial screening of article titles and abstracts, followed by full-text assessments based on predefined inclusion and exclusion criteria. The selection process was documented using a PRISMA flow diagram, ultimately yielding nine publications for inclusion in this review.

**Data Extraction:** A standardized form was used to capture essential information from each study, incorporating both publication and scientific details. To assess the credibility and academic reach of the articles, we carefully documented several journal-related attributes like journal name, whether it was indexed (e.g., in PubMed, Web of Science, or Scopus), and its standard (two-year) impact factor. We noted whether the journal employed single-blind, double-blind, or open peer review, or if any indication of peer-review rigor was provided. This offered insight into the quality control measures taken before publication. Disclosures about conflicts of interest were documented. We also extracted the article title, study ID, and the country in which the study was conducted. In terms of scientific content, we noted the biomarkers analyzed, the methods used, the aim of the study, and the overall design (including the total number of participants and any comparisons made).

**Data Synthesis and Analysis:** A narrative synthesis summarized and analyzed the extracted data. Studies were grouped thematically (e.g., prognostic value, predictive value, technical challenges). Different subtyping approaches and their clinical implications were described and critically appraised. Finally, knowledge gaps and the future research directions were identified.

## 3. Results

**Figure 2:**
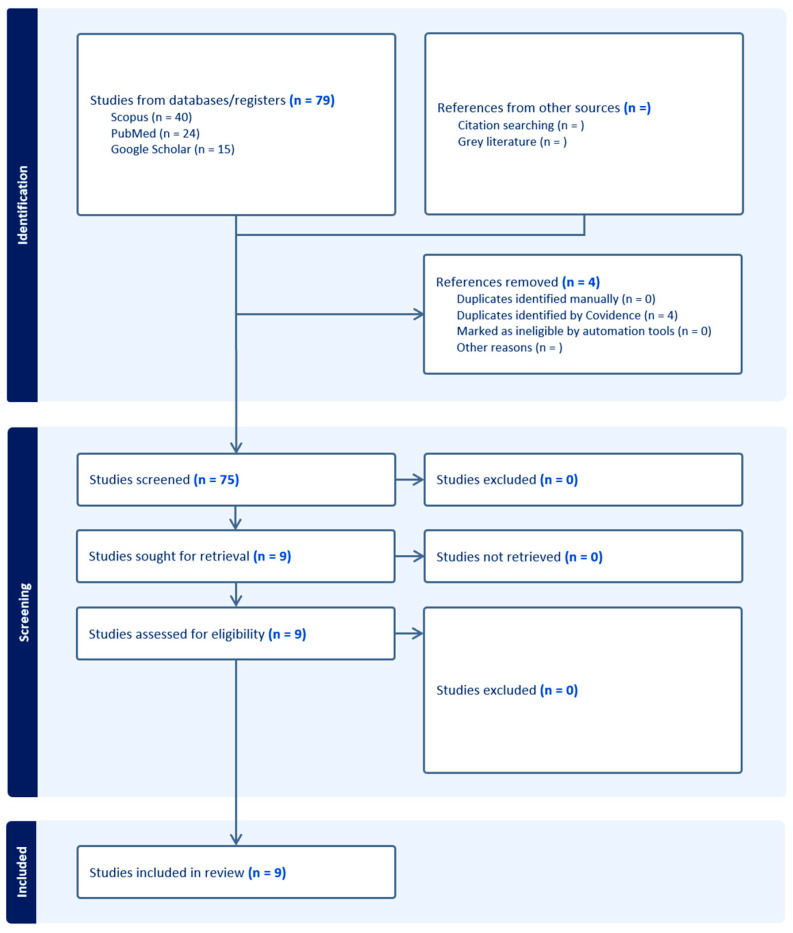
Flow diagram illustrates process of study identification, screening, and identification of studies for inclusion. Adapted from PRISMA flow diagram.

Seventy-nine studies were identified through our search strategy. Following deduplication, 75 studies remained and were screened by title, abstract, and full text, resulting in the inclusion of 9 retrospective studies. Figure 2 presents a flow diagram, adapted from the PRISMA flow chart, of the literature retrieval process.

Table 1 presents the biomarkers measured in the studies selected. The most frequently evaluated biomarker was GATA6, measured in four of the studies, followed by Keratin17 (KRT17) and Keratin81 (KRT81), which were included in two studies, respectively. Several studies investigated the potential of various biomarkers, including CREB3L1, GATA6, KRT81, HNF1A, and KRT17, to predict treatment response and prognosis. For instance, Xu et al. (2025) found that CREB3L1 expression was lower in patients who responded well to immunotherapy, suggesting its potential as a biomarker for treatment selection [28]. Similarly, studies exploring GATA6 as a prognostic marker indicated its association with enhanced immune surveillance and improved outcomes in certain PDAC subtypes. Other studies explored combinations of markers, such as KRT81 and HNF1A, to refine subtyping and predict responses to specific therapies like erlotinib and FOLFIRINOX. These findings highlight the potential of IHC-based molecular subtyping to personalize PDAC treatment.

Our scoping review identified several key predictive immunohistochemical (IHC) biomarkers for pancreatic ductal adenocarcinoma (PDAC). The review of studies highlights the critical role of predictive biomarkers in pancreatic ductal adenocarcinoma (PDAC) and their potential to enhance clinical outcomes. PDAC is characterized by its aggressive nature and poor prognosis, with a median overall survival (OS) of approximately 10–12 months for advanced stages and up to 28 months for resected cases with optimal adjuvant chemotherapy [33]. Recent research has identified various molecular subtypes of PDAC, with classifications based on gene expression patterns correlating with treatment responses and prognostic outcomes. These subtypes, such as the classical and basal-like groups defined by Moffitt et al. [34] and the quasimesenchymal and squamous subtypes identified by Collisson [15] and Bailey [35], offer potential for personalized therapy. For instance, the basal-like subtype is associated with poorer prognosis and chemoresistance. While promising, molecular subtyping has limitations. Gene expression can vary within a tumor, and the chosen technology may not capture all relevant molecular features, potentially leading to misclassification and hindering clinical translation [13,36,37].

Specific biomarkers such as KRT17 [12,38,39], GATA6 [26], HNF1A, and KRT81 [27] have emerged as significant predictors of clinical outcomes. GATA6, for example, has been shown to correlate positively with classical phenotype markers and negatively with basal-like markers in treatment-naive tumor [25]. The expression of KRT81 and HNF1A has also been linked to patient stratification, with implications for treatment response to therapies like erlotinib [27]. Briefly, IHC subtyping based on KRT81 and HNF1A effectively stratified PDAC into clinically relevant subgroups, with HNF1A expression correlating with superior overall and disease-free survival compared to KRT81. These results underscore the importance of identifying molecular subtypes and biomarkers to optimize patient stratification and guide individualized treatment strategies. Moreover, the role of KRT17 as a prognostic and predictive biomarker in pancreatic ductal adenocarcinoma (PDAC) sheds significant light on its potential utility in clinical settings. The identification of KRT17 as not only a marker of prognosis but also a predictive factor for treatment response represents a pivotal advancement in our understanding of PDAC pathology [12]. This research supports the implementation of KRT17 as a companion diagnostic marker, providing critical therapeutic insights that could guide more personalized treatment plans [12]. By incorporating KRT17 into routine diagnostic assessments, clinicians could better predict disease course, tailor treatment modalities to individual patients, and ultimately improve clinical outcomes in PDAC. Such findings reinforce the necessity of integrating robust molecular markers in the management of complex cancers, highlighting a step forward in the evolution of personalized oncology.

**Figure 3:**
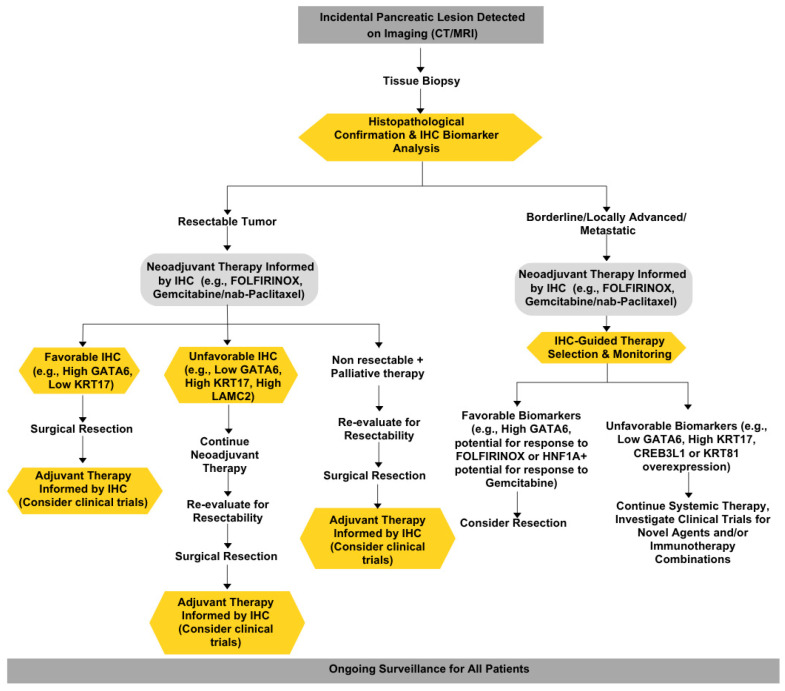
Proposed clinical management of pancreatic cancer patients.

Overall, these studies suggest that the integration of these biomarkers into clinical practice is crucial for enhancing patient management. The development of immunohistochemistry (IHC) assays for these biomarkers could facilitate their implementation in routine diagnostics, allowing for more personalized treatment approaches. However, these studies promptly reported that the retrospective nature of the analyses represented a limitation, suggesting that further prospective multi-center studies with larger patient populations and that the ongoing clinical trials evaluating the utility of these biomarkers in guiding treatment decisions in randomized controlled trials are needed [31,40]. The potential for these biomarkers to refine treatment selection and improve outcomes in PDAC is promising, but further studies are needed to establish their clinical applicability and effectiveness in diverse patient populations. In conclusion, the identification and validation of prognostic and predictive biomarkers in PDAC represent significant advancements in the quest for improved clinical outcomes. By enabling more precise patient stratification and personalized treatment strategies, these biomarkers have the potential to transform the management of this challenging malignancy (Figure 3).

### 3.1. The Utility and Clinical Imperative of IHC Biomarkers in PDAC

Mosele et al. (2024) highlight a crucial point: despite the theoretical promise of next-generation sequencing (NGS), its clinical utility in advanced PDAC remains limited in routine practice [41]. While NGS can identify a wide range of genomic alterations, only germline BRCA1/2 alterations currently hold ESCAT level I status (strong evidence of clinical actionability) in this setting. Although KRAS G12C mutations, detectable by PCR, provide a readily actionable target, they represent a small minority (1.5%) of PDAC cases [41,42]. This disparity between identifying molecular alterations and demonstrating improved patient outcomes underscores the central challenge—the “utility dilemma”. In contrast, immunohistochemistry (IHC), a technique used to visualize the presence and localization of specific proteins within a tissue sample, offers a pragmatic and readily implementable approach for assessing biomarker expression for several key reasons. IHC involves using labeled antibodies that bind specifically to the target protein. These antibodies are then detected through various methods, allowing for the visualization of the protein’s distribution within the tissue. Firstly, IHC is more cost-effective than more complex molecular techniques (e.g., gene expression profiling), making it a practical option for broader clinical use. Secondly, IHC is widely available in most pathology laboratories, requiring standard equipment and readily accessible reagents and thus eliminating the need for specialized infrastructure or highly trained personnel. This widespread availability facilitates easier integration into existing clinical workflows and diagnostic pathways. This is particularly important in developing countries, where the infrastructure for advanced molecular techniques is limited and expensive. Thirdly, IHC provides spatial context crucial for accurate interpretation, allowing for the visualization and assessment of protein expression within the tissue microenvironment and at the single-cell level. This is particularly important in heterogeneous tumors, where bulk molecular analyses may mask critical differences in expression patterns across distinct cell populations. Finally, for biomarkers whose regulation occurs predominantly at the translational rather than transcriptional level, IHC offers a more accurate representation of protein activity and functional status, directly measuring the protein product rather than the intermediary mRNA transcript. These combined advantages position IHC as a highly practical and readily deployable method that can be used for evaluating target biomarkers in routine clinical practice [43]. This rapid, readily available approach can inform timely treatment decisions, particularly crucial given the often-indiscriminate and potentially toxic “one-size-fits-all” approach to chemotherapy employed when treating metastatic PDAC [19]. By identifying patients most likely to benefit from specific treatments, based on IHC-detectable biomarkers, we can move towards a more personalized and effective approach to PDAC management, ultimately improving patient outcomes and minimizing harm.

### 3.2. The Utility Dilemma: Balancing Cost, Actionability, and Patient Benefit

The utility dilemma in PDAC centers on the challenge of balancing the potential benefits of advanced molecular diagnostics with the realities of cost, turnaround time, and clinical actionability. While the European Society for Medical Oncology (ESMO) guidelines recommend the use of tumor NGS within clinical genomics programs and trials, particularly for accessing PARP and NRG1 inhibitors [41], this recommendation is contingent on access to targeted therapies and local cost-effectiveness assessments. This highlights the inherent tension within the utility dilemma: the desire to provide cutting-edge diagnostics versus the responsible stewardship of healthcare resources. NGS, while offering a comprehensive genomic profile, is expensive, requires specialized infrastructure, and has long turnaround times, making it often incompatible with the rapid progression of PDAC. Furthermore, many identified alterations lack clear clinical actionability in the absence of readily available targeted therapies, diminishing the immediate patient benefit despite the high cost of testing. This raises the crucial ethical question: is it justifiable to expend significant resources on testing that yields limited actionable information, especially in a resource-constrained environment?

### 3.3. IHC: A Value-Based Solution to the Utility Dilemma

Immunohistochemistry (IHC) presents a strategically and fiscally responsible alternative to patient stratification in pancreatic ductal adenocarcinoma (PDAC) treatment. IHC is a technique that uses antibodies to detect specific proteins in tissue samples, allowing for the identification of biomarkers that can guide treatment decisions. In the US healthcare system, which is grappling with a trillion-dollar debt, the indiscriminate use of costly systemic chemotherapies, with often-limited efficacy in advanced PDAC [37], further exacerbates this financial burden. By using IHC to stratify patients based on their tumor biology, clinicians can select targeted therapies that are more likely to be effective, potentially minimizing the use of ineffective treatments and their associated costs and toxicities.

Compared to more expensive molecular profiling techniques like next-generation sequencing (NGS), IHC is cost-effective and widely available, making it a readily deployable solution for refining treatment selection. Its fiscal advantage is particularly relevant in the context of value-based healthcare, where the goal is to optimize patient outcomes while managing cost [36,44]. By directing resources towards targeted therapies that are more likely to benefit specific patient subgroups, IHC-guided treatment stratification can potentially improve the cost-effectiveness of PDAC care. In addition to its financial benefits, IHC also offers rapid results, often within 24 h with automated systems. This is a critical advantage given the aggressive nature of PDAC, where timely treatment initiation is crucial. The rapid turnaround time of IHC allows for prompt treatment decisions, maximizing the opportunity for intervention and aligning with the ethical imperative to avoid delays in care. In contrast, NGS results may take several weeks, potentially delaying treatment and compromising patient outcomes.

The current “one-size-fits-all” approach, particularly using FOLFIRINOX or gemcitabine/nab-paclitaxel without biomarker guidance, carries significant ethical baggage [21,45]. Subjecting patients to potentially unnecessary toxicity without a commensurate survival benefit raises serious ethical concerns. IHC-guided treatment selection has the potential to minimize these harmful effects, improving patient quality of life and reducing the burden on patients, caregivers, and families.

IHC has already demonstrated its value in guiding treatment decisions in other cancers. For example, in breast cancer, IHC is routinely used to assess estrogen receptor (ER), progesterone receptor (PR), and HER2 status [46]. Patients with ER/PR-positive tumors benefit from endocrine therapy [47], while those with HER2-positive tumors are candidates for targeted therapies like trastuzumab [48]. Similarly, in non-small-cell lung cancer, IHC is used to detect PD-L1 expression, which predicts the response to immune checkpoint inhibitors [49,50]. By tailoring treatment to the biology of each individual tumor, IHC has improved the outcomes and quality of life for patients with these cancers.

While NGS can identify alterations in 12–25% of PDAC cases [51,52], many lack immediate clinical utility [17]. IHC offers a pragmatic approach that can be used to address the utility dilemma by providing actionable information linked to established therapeutic strategies (31). Prioritizing IHC, therefore, not only aligns with financial responsibility but also upholds the ethical principles of beneficence and non-maleficence. A concerted effort to assess biomarkers with cost-effective methods like IHC is essential for realizing the tangible clinical benefits of precision oncology and ensuring equitable access to potentially life-saving diagnostic information [44].

### 3.4. Ethical Principles Guiding the Implementation of IHC Predictive Biomarkers

The ethical imperatives underlying pancreatic cancer care extend far beyond mere utility or efficiency. The current state of PDAC care, characterized by high mortality and limited treatment options, represents a significant challenge. This applies not just clinically, but ethically. The slow translation of research into clinical tools constitutes an ethical crisis demanding immediate action.

### 3.5. Autonomy

Patient autonomy requires individuals to make informed decisions about their medical care, which includes a comprehensive understanding of the purpose, implications, and limitations of, as well as alternatives to, IHC testing. This informed decision-making process is best achieved through shared decision-making, where clinicians and patients collaborate to make treatment choices aligned with the patient’s values and preferences [53]. IHC can facilitate this process by providing relatively easy-to-understand information compared to complex genomic data. However, clinicians must carefully present this information to ensure patient comprehension without causing information overload [54], particularly considering the difficult prognosis associated with PDAC and the emotional vulnerability of patients and their families. Maintaining patient trust is paramount, which necessitates transparency regarding the rationale for using IHC and interpreting its results [55]. Relying exclusively on “physician preference” to guide treatment decisions is unacceptable. Instead, physicians have a responsibility to engage in open communication with patients, actively discussing preferences and values, to ensure treatment recommendations reflect a genuinely shared decision-making process. [56]. Despite the benefits of IHC and shared decision-making, the application of patient autonomy in the context of novel diagnostics like IHC raises several important questions.

**(1) The Right to the “Best” vs. the Right to Choose:** Patient autonomy does not guarantee a right to the newest treatments or diagnostic tools simply because they exist. While patients have the right to make informed choices about their care, this right applies to available and appropriate options, considering factors such as cost, availability, and proven benefit. The principle of justice requires prioritizing interventions with established clinical utility over those with uncertain benefits or limited access, ensuring equitable resource allocation.

**(2) Autonomy, Privilege, and the Zero-Sum Game:** A “right” to the newest interventions for one patient might limit resources for others that are less privileged, highlighting the potential for privilege to influence autonomous decision-making. Prioritizing cost-effective, readily available tools like IHC becomes an ethical imperative to ensure equitable access to essential diagnostic information. Transparent communication about the benefits and limitations of different companion diagnostic approaches is crucial for empowering all patients to make informed decisions, regardless of socioeconomic status.

**(3) Shared Decision-Making**—Balancing Patient Input and Physician Expertise: Effective shared decision-making necessitates open communication and mutual respect. Physicians should provide clear information about available options, while patients share their values and preferences. In PDAC subtyping, this involves discussing the trade-offs between IHC and more complex molecular testing, considering factors such as cost, turnaround time, and clinical impact. While patient preferences are paramount, physicians have a responsibility to guide patients toward medically and ethically appropriate decisions based on the best available evidence [57]. The physician’s role extends beyond merely presenting options; it includes contextualizing those options within the framework of evidence-based medicine and ethical principles. This collaborative, evidence-based approach ensures personalized care while mitigating potential therapeutic misconceptions and upholding responsible resource allocation. It fosters trust by demonstrating transparency and a commitment to compassionate care and scientifically rigorous practice. The goal is to arrive at a decision that respects the patient’s autonomy while adhering to the physician’s responsibility to provide the best possible care based on the strongest available evidence.

### 3.6. Beneficence

The principle of beneficence dictates that healthcare providers should act in the best interests of their patients, maximizing benefits and minimizing potential harm [54]. IHC-based predictive biomarkers can promote beneficence by identifying the patients most likely to respond to specific therapies, improving treatment success rates, guiding the selection of less toxic therapies when appropriate, and potentially leading to earlier intervention. Furthermore, IHC can contribute to the development of more effective treatment strategies overall by facilitating the identification and validation of new therapeutic targets. However, the pursuit of beneficence must be tempered by a realistic assessment of the limitations of IHC and the potential for false-positive or false-negative results. Moreover, the scientific community must be vigilant against the unequal advancement and inclusion of biomarkers, a practice that can skew the landscape of available diagnostic and therapeutic options. This inequality often arises not purely from scientific need or clinical urgency but from a milieu influenced by publication pressures, funding opportunities, and the pursuit of prestige. These dynamics can cultivate an environment where certain biomarkers are disproportionately highlighted, not for their clinical utility but for their novelty and the academic currency they provide. This brings to light the concern of “self-promoted experts” who, perhaps driven by the need for career advancement, might disproportionately endorse specific biomarkers. Such endorsements can perpetuate a cycle where biomarkers are pursued aggressively at the expense of others, potentially sidelining equal or more effective alternatives that lack similar scientific or commercial backing. To navigate this complex landscape, it is essential for the medical community to foster a robust framework for validation and replication, emphasizing transparency and integrity in research. Further, engagement in multidisciplinary discourse and critique should be encouraged, ensuring that decisions around the adoption of specific IHC markers in PDAC are grounded in a broad consensus rather than the narrow interests of a few. It is only through rigorous scholarly examination and strict adherence to ethical standards that we can ensure the pursuit of beneficence in medical practice is genuinely fulfilled, thereby advancing patient care in both effective and morally responsible ways [58].

### 3.7. Non-Maleficence

This principle, often summarized as “do no harm”, requires healthcare providers to avoid causing unnecessary harm to patients. Given the known complexity and heterogeneity of PDAC, we and others have advocated for the inclusion of companion diagnostic markers that add predictive value in the clinical management of patients from the time of PDAC diagnosis (Figure 2) [12,59]. For example, the high expression of the transcription factor GATA6 and low expression of KRT17 have been associated with better responses to FOLFIRINOX chemotherapy in PDAC patients. In contrast, low GATA6 expression has been linked to a poor response to FOLFIRINOX but potentially better outcomes with gemcitabine-based regimens [25]. By incorporating these IHC biomarkers into treatment decision-making, clinicians can select therapies that are more likely to be effective based on the molecular profile of each patient’s tumor. This approach not only maximizes the potential for therapeutic benefit but also minimizes the risk of exposing patients to ineffective and potentially harmful treatments. It is important to note that while IHC itself is a minimally invasive procedure with minimal direct risks to patients, the potential harm lies in the misinterpretation or misapplication of IHC results, which could lead to suboptimal treatment choices. Therefore, it is crucial that IHC biomarker testing is performed in accredited laboratories with rigorous quality control measures and interpreted by experienced pathologists to ensure accurate and reliable results are obtained [45]. As our understanding of PDAC biology continues to evolve, the use of IHC biomarkers to guide treatment selection will become increasingly important in upholding the principle of non-maleficence and providing the best possible care for PDAC patients.

### 3.8. Justice

The principle of justice demands the fair and equitable distribution of healthcare resources. This is particularly relevant to the implementation of IHC biomarkers in PDAC, given the existing disparities in access to advanced cancer care [60]. IHC’s lower cost and wider accessibility, including in resource-limited settings, ensure that more patients can benefit from personalized treatment strategies. This is crucial in PDAC, where socioeconomic factors significantly influence access to care and outcomes. In contrast, expensive molecular tests like NGS may only be available in specialized centers or to patients with comprehensive insurance coverage or financial resources [61]. This disparity can create a two-tiered system, where some patients have access to cutting-edge molecular profiling while others are left with a one-size-fits-all approach. Prioritizing IHC as a cost-effective tool for patient stratification can help to close the treatment disparity gap and ensure that all patients, regardless of financial status or location, have the opportunity to receive tailored therapies based on their tumor’s molecular profile.

Moreover, IHC’s technical simplicity compared to NGS allows for more widespread adoption across different healthcare settings, including community hospitals and clinics. This decentralization of diagnostic capabilities can bridge the access gap between academic medical centers and local healthcare providers, enabling more patients to receive personalized care closer to home [62]. This is especially relevant for PDAC patients, who may face challenges related to transportation, time off work, and caregiver responsibilities when seeking care at distant, specialized centers. While IHC may serve as an interim solution, it facilitates more widespread treatment as we continue to progress towards discovering a definitive cure—something every patient rightfully deserves. However, the centralization of advanced molecular technologies in specialized centers and the lack of sociodemographic diversity among cancer clinical trial participants represent major barriers to advancing cancer care for the entire patient population [63]. Health systems often lack the specialized bioinformatics capacity required to analyze and interpret the complex data generated by tumor sequencing [64]. Despite evidence demonstrating improved outcomes in high-volume centers, a significant proportion of PDAC patients are diagnosed and treated in community hospitals with limited access to advanced molecular technologies and/or resources to use the information, highlighting the disparity in access related to the location of treatment [65]. To truly honor the spirit of medical innovation and ethical integrity, we must foster strategies that embrace the diversity of our society and advance equity within clinical trials, meticulously balancing the fine line between individual care and collective advancements in health sciences [66]. This commitment will ensure that our pursuit of knowledge remains just, transparent, and fundamentally aligned with the enduring welfare of humanity. By leveraging the equity-promoting characteristics of IHC, we can work towards a more just and inclusive approach to precision medicine in PDAC, ensuring that all patients have access to the diagnostic tools and tailored therapies that can improve their outcomes and quality of life. This commitment to reducing access disparities aligns with the ethical principle of justice, which calls for the fair distribution of healthcare resources and the elimination of unjust barriers to care.

## 4. Discussion

The pursuit of improved outcomes in pancreatic ductal adenocarcinoma (PDAC) necessitates a multi-faceted approach, and transcriptomic subtyping holds significant promise in this endeavor. As outlined in the preceding sections, large-scale, multi-center, and interdisciplinary efforts are crucial. Initiatives like COMPASS, PREDICT-PACA, Precision Promise, and others represent critical steps toward developing more effective, personalized treatment regimens. The PRIMUS-002 trial, performed as part of the PRECISION-Panc platform, exemplifies a prospective approach, integrating genomic and transcriptomic data with circulating biomarkers and clinical outcomes. This holistic approach, incorporating preoperative biopsies and resection specimens, is essential for understanding the dynamic interplay between molecular subtypes, therapeutic responses, and tumor heterogeneity. Critically, biomarker-based preoperative trials are paramount for validating predictive factors, moving beyond retrospective analyses to directly inform clinical decision-making.

However, the situation is further complicated by the scarcity of biomarker-driven clinical trials for PDAC patients, the inherent difficulties in conducting adequately powered trials in small molecular subgroups, and the practical considerations of turnaround time, cost-effectiveness, and reimbursement for molecular analyses. Innovative platforms like PRECISION-Panc, EPPIC, and Precision Promise represent potential solutions, integrating discovery, preclinical development, and innovative clinical trial design. The PRECISION-Panc Master Protocol, with its focus on EUS-guided biopsies and peripheral blood sampling, demonstrates a pragmatic approach to real-world personalized clinical trials, achieving a high success rate in molecular profiling.

## 5. Ethical Considerations: Utility, Equity, and Transparency

The advancement of transcriptomic subtyping and biomarker-driven approaches in PDAC is not solely a scientific endeavor; it is deeply intertwined with ethical considerations. Several key tensions emerge when analyzing this progress through the lens of ethical principles:

**Utility vs. Equity:** The principle of utility, aiming for the greatest good for the greatest number, is central to the pursuit of precision medicine. While sophisticated molecular profiling offers the potential for significant benefit, its high cost, complexity, and limited availability creates disparities in access. This aligns with the observation that the high cost, complexity, and limited availability of advanced technologies create significant disparities in access, as not all patients or healthcare systems have the resources to implement them. Patients treated in specialized centers with access to cutting-edge technologies and clinical trials are more likely to benefit from these developments than those treated in resource-constrained settings. This disparity raises fundamental questions of justice and fairness. A truly ethical approach demands a commitment to ensuring that the benefits of precision medicine are accessible to all patients, regardless of their geographic location, socioeconomic status, or race/ethnicity. This necessitates the prioritization of more equitable solutions like IHC, which can provide similar benefits at a lower cost and with wider accessibility. Therefore, ongoing efforts are needed to address these underlying disparities such as geographic location and socioeconomic status and ensure fair access to healthcare resources. This requires innovative strategies to reduce costs, decentralize expertise, and expand access to clinical trials. For instance, implementing reimbursement policies that cover the cost of molecular profiling and providing funding for research and development to improve the efficiency and affordability of these technologies are essential steps. Additionally, establishing guidelines and standards for use in clinical practice can also help streamline access. As Varkey et al. point out, even when treating physicians act with knowledge, skill, and empathy, treatment selection may be made without absolute certainty about effectiveness, highlighting the need for therapies established on a rigorous scientific foundation that is accessible to all [54].

**Utility vs. Transparency:** The principle of utility is intrinsically linked to transparency in research, demanding openness in data, protocols, and processes to ensure the validation of findings and foster scientific advancement. Transparency’s absence, characterized by data hoarding and replication difficulties, obstructs progress and compromises research ethics. A shift towards open science, emphasizing rigorous hypothesis testing over mere confirmation, is paramount. This requires the use of freely accessible methodologies, protocols, raw data, software, and code. Critically, procedural justice necessitates the careful examination of who defines utility, access, and associated trade-offs. The meaningful engagement of key stakeholders, representing the diverse voices of the public, is vital. Such inclusive dialogue ensures that the pursuit of utility does not disproportionately favor some at the expense of others. Deliberate efforts to incorporate diverse perspectives into scientific governance and decision-making are essential. Neglecting these considerations risks inequitable outcomes, undermining the very utility that transparency aims to promote. This highlights the need for greater equity within the realm of open science itself.

**Internal Tensions within Utility (Cost–Benefit Analysis): Tensions even arise** within the principle of utility itself. The pursuit of precision medicine involves significant costs—financial, technological, and human. A rigorous cost–benefit analysis is essential, weighing the potential benefits of improved outcomes against the resources required. This analysis must consider not only the direct costs of molecular profiling and targeted therapies but also the indirect costs associated with infrastructure, training, and data management. A purely utilitarian perspective would suggest that the most cost-effective strategies that benefit the most patients should be prioritized. That could mean prioritizing basic research and widely applicable, less expensive screening methods (like IHC) instead of more complex and individualized molecular tests.

**Biomarker-Based vs. Biomarker-Agnostic Strategies:** Navigating the future of PDAC care requires careful consideration of both biomarker-based and biomarker-agnostic approaches. While targeted therapies based on specific mutations can be highly effective, many patients either lack access to comprehensive molecular profiling or do not present with actionable mutations. In these instances, readily available approaches such as IHC (immunohistochemistry) become vital. Moving forward, a comprehensive strategy that integrates both biomarker-based and biomarker-agnostic treatments will help ensure that all PDAC patients receive appropriately tailored care. Previously, next-generation sequencing (NGS) was employed to pinpoint the “needle in the haystack”—roughly 5% of patients with microsatellite instability or DNA damage response mutations. This was the group that could benefit from immunotherapy or PARP inhibitors [67]. Although the cost–benefit ratio justified NGS for this select group, emerging evidence suggests that broader sequencing may be less crucial for most patients. For example, results from clinical trials of RevMed’s RAS-ON inhibitors (RMC-6236 and RMC-7977)—which targets both mutant and wild-type KRAS—hold exceptional promise and could revolutionize PDAC therapy if approved. In contrast, mutant-specific KRAS inhibitors (e.g., MRTX1133 G12Di) have not shown similarly positive outcomes, leaving NGS screening relevant primarily for that small subgroup with actionable mutations [68,69]. Looking ahead, the critical question is determining who stands to gain the most from RAS-ON inhibitors once standard-of-care chemotherapy (e.g., gemcitabine/nab-paclitaxel or FOLFIRINOX) loses effectiveness. Recent findings by Dilly et al. suggest that basal like tumors—easily identified through the IHC detection of surrogate markers such as KRT17—are especially responsive to these agents [70]. This highlights the pivotal role of IHC-based testing in guiding both first-line therapy and second-line decisions regarding RAS-ON inhibitors. As a result, expanding IHC use has the potential to enhance clinical decision-making, ensuring that emerging treatments are deployed for those most likely to benefit.

By examining these aspects, we can identify the best practices and develop strategies to accelerate the translation of research findings into clinical benefit for all PDAC patients.

## 6. Conclusions

In line with prior research, our study underscores the vital necessity of advanced, ethically informed strategies for managing pancreatic ductal adenocarcinoma (PDAC). By integrating early molecular classification into treatment protocols, we can enhance patient outcomes, reflecting the critical importance of timely and strategic interventions. To fulfill this promise, it is imperative that we foster broad-based collaboration among diverse stakeholders—ranging from policymakers to academicians and industry experts—to universally advance access to predictive biomarkers and precision medicine. This concerted effort aims to bridge clinical and socioeconomic gaps but also to propel society into a new era of cancer treatment. Embracing such a holistic approach, underscored by a commitment to ethical principles and fueled by robust interdisciplinary cooperation, holds the key to transformative advances in PDAC therapy, ensuring that these innovations benefit patients globally and culminate in a future where the devastating impact of this disease is markedly diminished.

## Figures and Tables

**Table 1:** Characteristics and outcomes of published studies.

Journal Name	Journal Indexed	Impact Factor	Journal Peer Review Process	Conflict of Interest	Author	Title	Study ID	Country in Which the Study Conducted	Biomarker(s)	Methods	Aim of Study	Study Design	Total Number of Participants	Comparison	Outcome	Conclusion
Journal for ImmunoTherapy of Cancer	Yes	10.3	Single anonymized	None	Xu, 2025 [28]	CREB3L1 facilitates pancreatic tumor progression and reprograms intratumoral tumor-associated macrophages to shape an immunotherapy-resistance microenvironment	https://doi.org/10.1136/jitc-2024-010029	China	CREB3L1	IHC	Explore CREB3L1’s role in PDAC and its correlation with clinical features	Cohort study	94	Responders (ORR) vs. non- ORR; IHC-based CREB3L1 expression	Lower CREB3L1 expression found in those responding well to immunotherapy	CREB3L1 expression predicts immunotherapy response and outcome in PDAC
Cell Reports Medicine	Yes	11.7	Single anonymized	None	Van Eijck, 2024 [29]	GATA6 identifies an immune-enriched phenotype linked to favorable outcomes in patients with pancreatic cancer undergoing upfront surgery	https://doi.org/10.1016/j.xcrm.2024.101557	Other: the Netherlands	GATA6	Transcriptomic profiling/IHC	Investigate prognostic value of GATA6 IHC in treatment-naive vs. gemcitabine-based nCRT-treated PDAC patients	Cohort study	88	GATA6 expression, immune cell infiltration, and survival in treatment-naive vs. chemotherapy-treated patients	Strongly treatment-naive GATA6 tumors exhibit enhanced immunological features and immunostimulatory mechanisms	GATA6 expression correlates with distinct immune landscapes and better outcomes, underscoring its prognostic significance
The Journal of Pathology: Clinical Research	Yes	3.4	Single anonymized	None	Rao, 2024 [27]	KRT81 and HNF1A expression in pancreatic ductal adenocarcinoma: investigation of predictive and prognostic value of immunohistochemistry-based subtyping	https://doi.org/10.1002/2056-4538.12377	Other: Germany	KRT81 and HNF1A	IHC	Validate revised subtyping in a controlled phase III trial (CONKO-005) assessing erlotinib + gemcitabine vs. gemcitabine	Cohort study	269	Gemcitabine–erlotinib combination vs. gemcitabine only	HNF1A-positive subtype benefited more from gemcitabine–erlotinib compared with KRT81-positive or double-negative subtypes	KRT81/HNF1A IHC serves as a partial surrogate for RNA-defined PDAC subtypes and informs potential treatment choices
Clinical Cancer Research	Yes	10.4	double-blind review	A. Stenzinger reports receiving speaker’s bureau honoraria from AstraZeneca, Illumina, Novartis, and Thermo Fisher Scientific and is a consultant/advisory board member for AstraZeneca, Bristol-Myers Squibb, Illumina, Novartis, and Thermo Fisher Scientific. M.R. Sprick is listed as a co-inventor on a pending patent application on the use of KRT81 and HNF1A for the stratification of PDAC patients. This is owned by HI-STEM gGmbH. No potential conflicts of interest were disclosed by the other authors.	Muckenhuber, 2018 [30]	Pancreatic Ductal Adenocarcinoma Subtyping Using the Biomarkers Hepatocyte Nuclear Factor-1A and Cytokeratin-81 Correlates with Outcome and Treatment Response	https://doi.org/10.1158/1078-0432.CCR-17-2180	Other: Germany	HNF1A and KRT81	IHC	Validate HNF1A/KRT81 subtypes in resectable and unresectable PDAC, and assess their predictive value for chemotherapy	Cohort study	204	Survival and treatment response (FOLFIRINOX vs. gemcitabine) by HNF1A/KRT81 IHC subtypes	KRT81-positive patients derived less benefit from FOLFIRINOX; HNF1A-positive patients had a significantly better initial response to FOLFIRINOX compared with gemcitabine	An IHC-based HNF1A/KRT81 classification reliably identifies PDAC subtypes, aiding pre-therapeutic stratification and personalized therapy
Translational Research	Yes	6.4	Single anonymized	MFB has received research funding from Celgene, Frame Therapeutics, and Lead Pharma, and has acted as a consultant to Servier and Olympus. HWML Consultant or advisory role: BMS, Daiichy, Dragonfly, Eli Lilly, MSD, Nordic Pharma, Servier. Research funding and/or medication supply: Bayer, BMS, Celgene, Janssen, Incyte, Eli Lilly, MSD, Nordic Pharma, Philips, Roche, Servier. Speaker role: Astellas, Daiichy, Novartis. JPM has acted as a consultant to AbbVie. JWW Consultant or advisory role: MSD, Servier, Astra Zeneca, Research funding: MSD, Nordic, Servier. Speaker role: MSD, Servier. None of these parties were involved in the design of this study or drafting of the manuscript. All other authors declare no conflicts of interest.	Lansbergen, 2024 [31]	Transcriptome-based classification to predict FOLFIRINOX response in a real-world metastatic pancreatic cancer cohort	https://doi.org/10.1016/j.trsl.2024.08.002	Other: the Netherlands	GATA6 and keratin-17 (KRT17)	IHC	Assess predictive value of molecular subtypes for FOLFIRINOX response in advanced PDAC	Cohort study	86	IHC-based stratification into good vs. poor responders to FOLFIRINOX	GATA6 H-score reliably predicts response to FOLFIRINOX	GATA6 (±KRT17) IHC can be integrated into diagnostic routines to guide chemotherapy choice and inform patient prognosis
Scientific Reports	Yes	3.8	Single anonymized	The authors declare no competing interests.	Duan, 2021 [25]	The value of GATA6 immunohistochemistry and computer-assisted diagnosis to predict clinical outcome in advanced pancreatic cancer	https://doi.org/10.1038/s41598-021-94544-3	Canada	GATA6	IHC	Evaluate GATA6 IHC as a single biomarker for advanced PDAC and examine computer-assisted analysis	Cohort study	110	Response to chemotherapy according to GATA6 IHC	Among mFFX-treated patients, disease progression was 39% in GATA6-low vs. 12% in GATA6-high PDAC	GATA6 IHC can serve as a single predictive biomarker for mFFX response in advanced PDAC; warrants prospective validation
American Journal of Clinical Pathology	Yes	5.4	double-blind review	K.R.S. and L.F.E.-H. are consultants for KDx Diagnostics. E.M.B. is an employee of Perthera and owns stocks in the company. He also has consulted for Theralink Technologies and received compensation as chair of the Science Advisory Board and owns stock in the company. The additional authors have nothing to disclose.	Delgado-Coka, 2024 [12]	Keratin 17 is a prognostic and predictive biomarker in pancreatic ductal adenocarcinoma	https://doi.org/10.1093/AJCP/AQAE038	United States	Keratin 17	IHC	Define K17 IHC thresholds for chemotherapy response to optimize therapeutic interventions in PDAC	Cohort study	305	K17 expression in the context of survival after adjuvant chemotherapy	High K17 marks resistance to gemcitabine-based therapies and predicts better response to 5-FU-based regimens	K17 IHC testing serves as a rapid predictive marker to guide the best chemotherapy choice based on tumor expression profiles
Scientific Reports	Yes	3.8	Single anonymized	CI-D receives research support from BMS. All other authors (TS, AH, MT, K-Ki, YJH, K-Ka, TY, SK, Ko-K, KO, KG, NO, JM, YS, TH, JS) declare that they have no conflicts of interest in relation to this work.	Shibayama, 2024 [26]	Combination immunohistochemistry for CK5/6, p63, GATA6, and HNF4a predicts clinical outcome in treatment-naive pancreatic ductal adenocarcinoma	https://doi.org/10.1038/s41598-024-65900-w	Other: Japan	CK5/6, p63, GATA6, and HNF4a	Immunohistochemistry (IHC) on endoscopic ultrasound-guided fine-needle aspiration biopsy (EUS-FNAB) samples	Determine surrogate biomarkers (K5/6, p63, GATA6, HNF4a) for molecular signatures in advanced PDAC	Cohort study	190	IHC-based subtypes (Classical, Transitional, Basal-like)	Basal-like pattern correlated with squamous differentiation and worst survival; (Transitional/Basal vs. Classical) = poor prognosis	IHC expression patterns identify Basal-like PDAC, aiding in prognostic assessment and therapeutic decision-making.
Cancers	Yes	5.2	Single anonymized	Authors declare no conflict of interest	Okada, 2021 [32]	Identification of LAMC2 as a prognostic and predictive biomarker for determining response to gemcitabine-based therapy in pancreatic ductal adenocarcinoma	https://doi.org/10.1016/j.ejca.2020.12.031	Japan	LAMC2 (Laminin γ2)	IHC on EUS-FNAB samples.	Identify and validate biomarkers for gemcitabine-based therapy response in PDAC	Retrospective analysis of datasets and clinical cohorts	423	LAMC2 expression compared with overall survival, relapse-free survival, and gemcitabine response	High LAMC2 correlates with poor prognosis and reduced response to gemcitabine	LAMC2 serves as a prognostic and predictive biomarker for gemcitabine-based therapies in PDAC
